# Antibiofilm Activities of Borneol-Citral-Loaded Pickering Emulsions against Pseudomonas aeruginosa and Staphylococcus aureus in Physiologically Relevant Chronic Infection Models

**DOI:** 10.1128/spectrum.01696-22

**Published:** 2022-10-04

**Authors:** Wen Wang, Xuerui Bao, Mona Bové, Petra Rigole, Xiaofeng Meng, Jianyu Su, Tom Coenye

**Affiliations:** a School of Food Science and Engineering, South China University of Technology, Guangzhou, Guangdong, China; b Laboratory of Pharmaceutical Microbiology, Ghent University, Ghent, Belgium; c China-Singapore International Joint Research Institute, Guangzhou, China; Louis Stokes Cleveland VAMC

**Keywords:** combination treatment, Pickering emulsion, biofilms, chronic infection, *Pseudomonas aeruginosa*, *Staphylococcus aureus*, borneol, citral

## Abstract

Phytochemicals are promising antibacterials for the development of novel antibiofilm drugs, but their antibiofilm activity in physiologically relevant model systems is poorly characterized. As the host microenvironment can interfere with the activity of the phytochemicals, mimicking the complex environment found in biofilm associated infections is essential to predict the clinical potential of novel phytochemical-based antimicrobials. In the present study, we examined the antibiofilm activity of borneol, citral, and combinations of both as well as their Pickering emulsions against Staphylococcus aureus and Pseudomonas aeruginosa in an *in vivo*-like synthetic cystic fibrosis medium (SCFM2) model, an *in vitro* wound model (consisting of an artificial dermis and blood components at physiological levels), and an *in vivo* Galleria mellonella model. The Pickering emulsions demonstrated an enhanced biofilm inhibitory activity compared to both citral and the borneol/citral combination, reducing the minimum biofilm inhibitory concentration (MBIC) values up to 2 to 4 times against P. aeruginosa PAO1 and 2 to 8 times against S. aureus P8-AE1 in SCMF2. In addition, citral, the combination borneol/citral, and their Pickering emulsions can completely eliminate the established biofilm of S. aureus P8-AE1. The effectiveness of Pickering emulsions was also demonstrated in the wound model with a reduction of up to 4.8 log units in biofilm formation by S. aureus Mu50. Furthermore, citral and Pickering emulsions exhibited a significant degree of protection against S. aureus infection in the G. mellonella model. The present findings reveal the potential of citral- or borneol/citral-based Pickering emulsions as a type of alternative antibiofilm candidate to control pathogenicity in chronic infection.

**IMPORTANCE** There is clearly an urgent need for novel formulations with antimicrobial and antibiofilm activity, but while there are plenty of studies investigating them using simple *in vitro* systems, there is a lack of studies in which (combinations of) phytochemicals are evaluated in relevant models that closely resemble the *in vivo* situation. Here, we examined the antibiofilm activity of borneol, citral, and their combination as well as Pickering emulsions (stabilized by solid particles) of these compounds. Activity was tested against Staphylococcus aureus and Pseudomonas aeruginosa in *in vitro* models mimicking cystic fibrosis sputum and wounds as well as in an *in vivo* Galleria mellonella model. The Pickering emulsions showed drastically increased antibiofilm activity compared to that of the compounds as such in both *in vitro* models and protected G. mellonella larvae from S. aureus-induced killing. Our data show that Pickering emulsions from phytochemicals are potentially useful for treating specific biofilm-related chronic infections.

## INTRODUCTION

Pathogenic biofilm-associated bacteria adhering to biotic or abiotic surfaces are a major challenge to food industries and health care. Staphylococcus aureus and Pseudomonas aeruginosa are commonly involved in biofilm-associated infections, including wound infections, lung infections in cystic fibrosis (CF) patients, and implant-related infections. Evidence suggests that bacteria in such infections usually grow as spatially organized micrometer-sized, dense biofilm aggregates that show reduced susceptibility to antimicrobials, and there is an urgent need for effective antibiofilm treatments (1–3).

**FIG 1:**
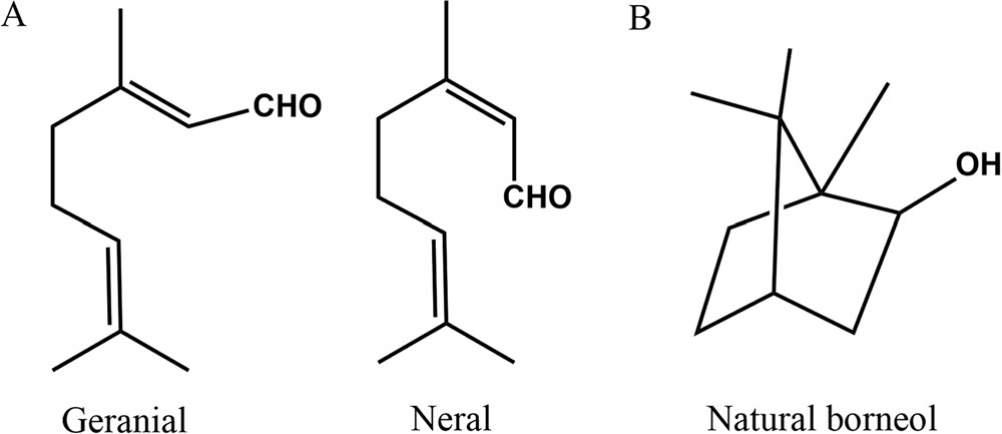
Two isomers of citral, geranial (trans-citral) and neral (cis-citral) (A), and chemical structure of natural borneol (B).

Previous experiments have demonstrated that citral combined with borneol has a broad-spectrum antibacterial effect that can be attributed to a disruption of bacterial membrane integrity (4). Citral, the predominant component in Litsea cubeba essential (5) and lemongrass (Cymbopogon citratus) oils (6), is a natural isoprene compound and a representative of open-chain monoterpenes with lemon flavor (7). It is a natural mixture of the following two isomeric acyclic monoterpene aldehydes (C_10_H_16_O): geranial (*trans*-citral) and neural (*cis*-citral) (Fig. 1A). Previous studies have shown that citral can increase the glucose and malondialdehyde content of cells and induces protein leakage by damaging the bacterial cell wall and membrane (8–10). Borneol (Fig. 1B) is a natural bicyclic terpene derivative with antibacterial and antiadhesive properties (11, 12). Because of its penetration-promoting effect (13–15), borneol is often used in combination with other antibacterial agents to exert a synergistic antibacterial effect (16–18). Citral and borneol are both oxygenated monoterpenes that can penetrate phospholipid membranes, physically disturbing their structural and functional properties (19). However, for the successful eradication of biofilms, the lipophilic volatile substances need to penetrate into the aqueous environment of the biofilm.

Pickering emulsions provide an effective route to encapsulate hydrophobic molecules within a self-assembled colloidal shell, which is stabilized by an insoluble solid. Attractive properties of these Pickering emulsions include reduced toxicity, enhanced stability, and a higher surface load and thickness compared to conventional surfactants (20, 21). The particles at the oil-water interface form a steric barrier, which can prevent the interface interaction and oil droplet aggregation through volume exclusion (22, 23). Inorganic nanoparticles, such as hydrophilic amine-functionalized silica nanoparticles (SiO_2_-NH_2_ NPs), can act as stabilizers for Pickering emulsions (24). For example, oil/water cinnamaldehyde/peppermint oil Pickering emulsions were fabricated by an *in situ* reaction at the oil/water interface between SiO_2_-NH_2_ NPs and cinnamaldehyde, which structurally augments the capsules to efficiently deliver the essential oil payloads, effectively eradicating clinically relevant bacterial biofilms (25).

The antibiofilm effect of borneol/citral (BC)-based Pickering emulsion stabilized by SiO_2_-NH_2_ NPs increased 2- to 4-fold compared with that of citral or borneol alone in conventional liquid or agar medium (4). However, in these studies, activity was determined using simple *in vitro* models (e.g., using stainless steel, glass, and polycarbonate as substrate for biofilm formation), and the conditions in these assays are very different from those *in vivo* at the site of infection. The goal of the present study is to evaluate the antibiofilm efficacy of the combination of borneol and citral and the relevant Pickering emulsions toward P. aeruginosa and S. aureus biofilms. The *in vivo*-like chronic infection models used are a synthetic cystic fibrosis medium (SCFM2) (26) and an *in vitro* wound model (consisting of an artificial dermis and blood components at physiological levels) (27). Finally, we also used the Galleria mellonella infection model to evaluate *in vivo* toxicity and antimicrobial efficacy.

## RESULTS AND DISCUSSION

### Pickering emulsions can effectively reduce the number of viable bacteria but cannot completely eliminate an established biofilm of P. aeruginosa PAO1 in SCFM2.

The borneol/citral combinations and Pickering emulsions were tested for their ability to inhibit biofilm formation by P. aeruginosa PAO1 in the SCFM2 medium. As there was a significant difference in number of CFU recovered from untreated controls and from biofilms exposed to solvent (4.75 to 12% ethanol) alone (*P* < 0.0001) (see Fig. S1A in the supplemental material), all experimental groups were compared to controls containing the same concentration of ethanol. Borneol alone was able to reduce the number of viable bacteria by 1.3 log units at a concentration of 2.5 mg/mL (Fig. S1), while at the same concentration citral alone was only able to reduce the number of viable bacteria by 0.3 log units. At a concentration of 10 mg/mL, borneol or citral alone lowered the number of CFU to below the detection limit (10 CFU/mL) (Fig. S1). Considering the inherent low solubility of these two phytochemicals (4) and in order to exclude the influence of the solvent (ethanol) on biofilm formation, the subsequent antibiofilm activity studies were only carried out with the combinations of BC (1:9) and BC (2:8). BC (2:8) at a concentration of 5 mg/mL reduced the bacterial count by 1.9 log units compared to that of the solvent control. BC (1:9) and BC (2:8) at a concentration of 10 mg/mL completely inhibited the growth of P. aeruginosa PAO1 in SCFM2 (Fig. S1). The minimum biofilm inhibitory concentration (MBIC) values of borneol, citral, BC (1:9), and BC (2:8) were 2.5, 10, 10, and 5 mg/mL, respectively (Table 1). Compared to the untreated control, all Pickering emulsions inhibited bacterial growth by at least 90% at concentrations between 2.5 and 10 mg/mL but were unable to completely kill all bacteria.

**TABLE 1:** MBIC, MBEC_90_, and MBEC values of different treatments for P. aeruginosa PAO1 and S. aureus P8-AE1 determined in SCFM2

Treatment	MBIC^*a*^ (mg/mL)		MBEC_90_^*c*^ (mg/mL)		MBEC^*b*^ (mg/mL)	
	PAO1	P8-AE1	PAO1	P8-AE1	PAO1	P8-AE1
Borneol	2.5	1	>20	0.5	>20	>4
Citral	10	0.5	>20	0.5	>20	4
C-Cap	2.5	0.25	1.25	0.125	>20	1
BC (1:9)	10	0.5	10	0.5	20	1
BC-Cap (1:9)	2.5	0.0625	1.25	0.125	>20	1
BC (2:8)	5	0.5	2.5	0.5	20	0.5
BC-Cap (2:8)	2.5	0.25	1.25	0.125	>20	0.5

a MBIC, minimum biofilm inhibitory concentration, the lowest concentration of antibacterial agent that inhibited biofilm growth by at least 90% compared to that of the untreated control.

b MBEC, minimal biofilm eradication concentration, the lowest concentration of antibacterial agent that prevents visible growth on the TSA plate after 24 h of incubation recovered from preformed biofilm.

c MBEC_90_, the lowest concentration of antibacterial agent that killed at least 90% of bacteria in preformed biofilm (1-log-unit difference) compared to the control.

We also assessed the Pickering emulsion’s activity against 24-h-old biofilms. We first confirmed that P. aeruginosa PAO1 formed biofilm aggregates in SCFM2 in the conditions used in the present study, and also a large amount of green viscous flocculent mucus was observed in 24-h performed P. aeruginosa PAO1 biofilm (see Fig. S3 in the supplemental material). The bacterial density was approximately 2.8 × 10^9^ CFU/mL. Only high concentrations of BC (1:9) and BC (2:8) (i.e., 10 mg/mL) resulted in a significant reduction in the number of culturable cells (CFU/mL) recovered from a 24-h P. aeruginosa PAO1 biofilm grown in SCFM2, but at these concentrations, the solvent alone also had a profound effect, complicating the interpretation of these data (see Fig. S2A in the supplemental material). Pickering emulsions were able to decrease the number of cells recovered from the biofilm by approximately 1.1 log units at a concentration of 1.25 mg/mL (*P* < 0.001) compared to that from the untreated control (see Fig. 3A). Compared to citral alone and the combinations, the minimal biofilm eradication concentration that killed at least 90% of bacteria in preformed biofilm (MBEC_90_) values of Pickering emulsions decreased 16- (citral-loaded pickering [C-Cap] emulsion), 8- (borneol-citral-loaded pickering [BC-Cap] emulsion), and 2-fold (BC-Cap, 2:8), respectively (Table 1). However, none of these Pickering emulsions could completely eliminate all of the bacteria from the preformed biofilms.

**FIG 2:**
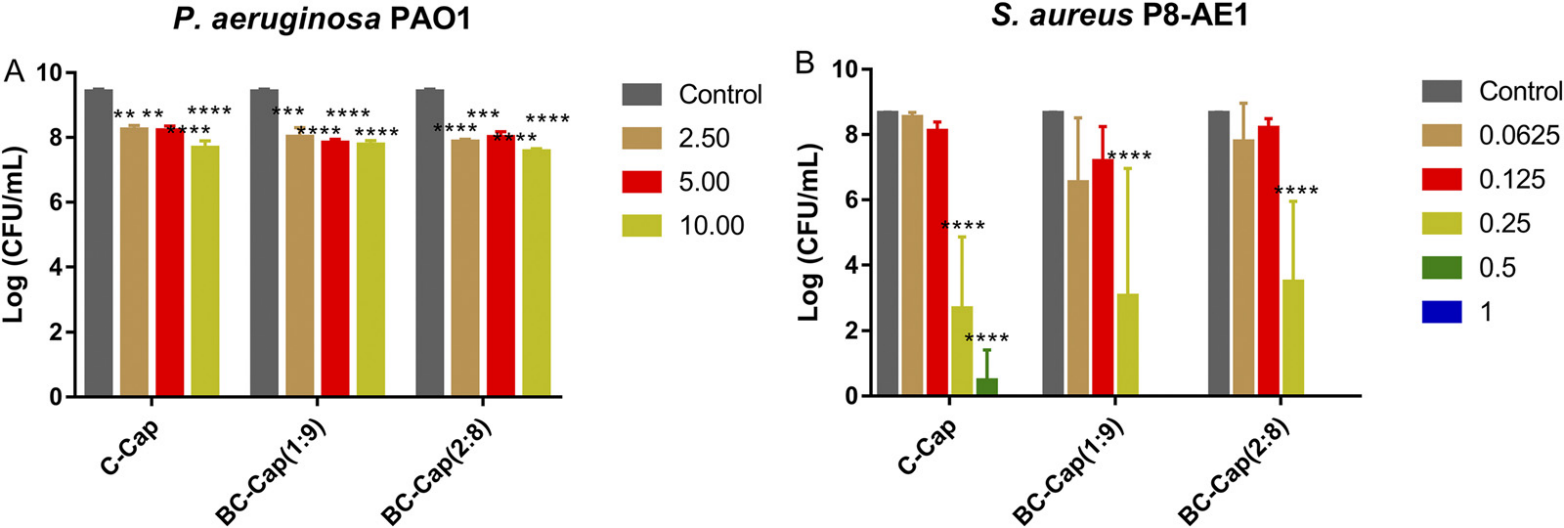
Biofilm inhibitory activities of Pickering emulsions (mg/mL) against P. aeruginosa PAO1 and S. aureus P8-AE1 in SCFM2 medium after 24 h of incubation. Inhibition activity was assessed by CFU count. Control represents untreated bacteria. The detection limit is 10 CFU/mL. Data are reported as mean ± standard deviation. *P* values obtained by one-way ANOVA followed by Tukey test. *, *P* < 0.05; **, *P *< 0.01; ***, *P *< 0.001; ****, *P *< 0.0001.

**FIG 3:**
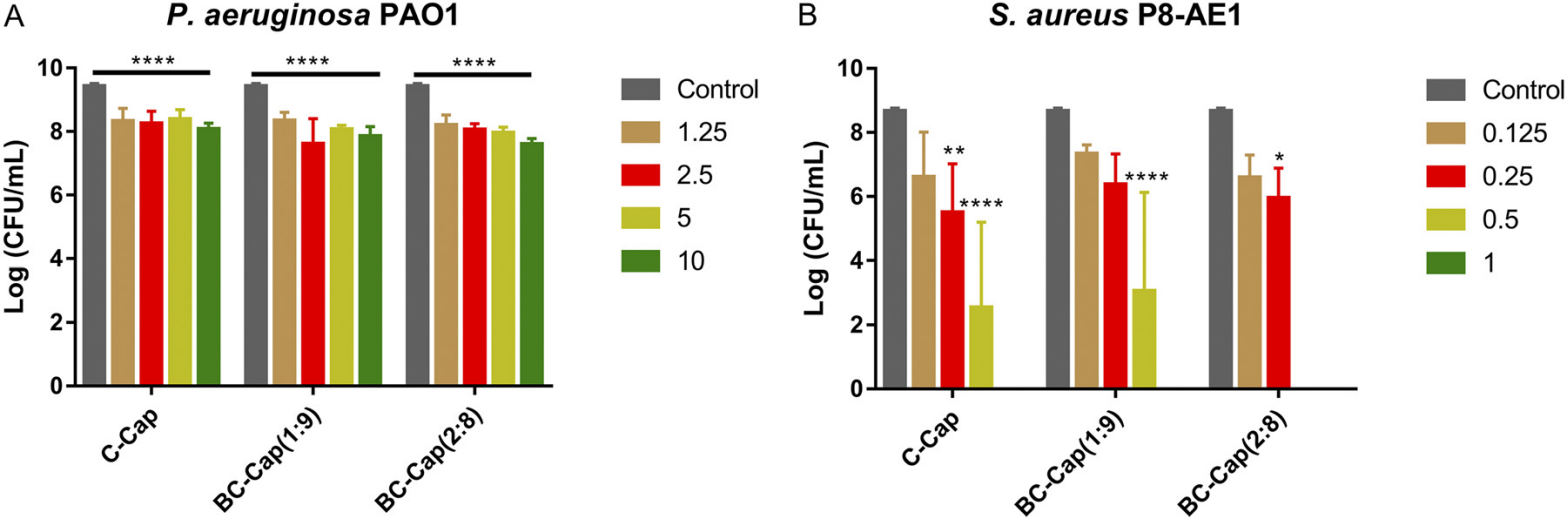
Number of culturable cells (CFU/mL) recovered from 24-h P. aeruginosa PAO1 biofilm and S. aureus P8-AE1 biofilm grown in SCFM2 after exposure to different concentrations of Pickering emulsions (mg/mL). Control represents untreated bacteria. Error bars represent the standard deviation. *, *P *< 0.05; **, *P *< 0.01; ***, *P *< 0.001; ****, *P* < 0.0001.

In conclusion, Pickering emulsions showed antibiofilm activity against P. aeruginosa in concentrations ranging from 1.25 mg/mL to 10 mg/mL. Recent data (28) demonstrated that lower concentrations (2 or 4 mg/mL) of citral only slowed down the bacterial multiplication within 5 h and that only 16 mg/mL citral completely inhibited bacteria growth within 18 h. Low concentrations of citral (2 or 4 mg/mL) were also confirmed to increase expression of efflux operons, namely, *mexAB*-*oprM* and *mexEF-oprN*, which are known to play a role in multiple resistance in various Pseudomonas species (29–31). In addition, P. aeruginosa has the ability to degrade terpenes (including citral and borneol) and to use them as a sole source of carbon and energy (32–34), which may be the reason why different concentrations of C-Cap and BC-Cap have similar antibiofilm activity against P. aeruginosa PAO1 (as shown in Fig. 2 and 3).

### Combination borneol/citral and their Pickering emulsions can effectively kill S. aureus P8-AE1 in biofilms in SCFM2.

We subsequently evaluated the inhibitory effect of combinations of borneol and citral and their Pickering emulsions on S. aureus P8-AE1 biofilm formation. For S. aureus P8-AE1, no effect of the solvent in concentrations up to 2.4% were observed. As shown in Fig. S1B, both borneol and citral could kill S. aureus P8-AE1 cells. Compared to 24-h-old biofilms, citral alone was able to reduce the number of viable bacteria by 3.2 log units at a concentration of 0.5 mg/mL. The MBIC of citral (0.5 mg/mL) (Table 1) against S. aureus P8-AE1 is almost identical to the previously reported MIC value (0.45 mg/mL) against methicillin-resistant S. aureus (35). Both borneol and BC (2:8) were able to reduce the number of viable bacteria by 6.4 log units at 1.0 mg/mL. Citral alone and the combination BC (1:9) at a concentration of 1.0 mg/mL resulted in the reduction of the initial bacterial inoculum below the limit of detection (i.e., below 10 CFU/mL). BC (1:9) at a concentration of 0.5 mg/mL exhibited powerful biofilm inhibitory effect (approximately 4.1 log units compared to the untreated group). Compared with unencapsulated phytochemicals, the Pickering emulsions led to an additional biofilm inhibition of 6.1 log units (C-Cap, 0.25 mg/mL), 2.2 log units (BC-Cap, 1:9, 0.0625 mg/mL), and 5.4 log units (BC-Cap, 2:8, 0.25 mg/mL) (Fig. 2B and Table 1; see also Fig. S1B).

The biofilm eradicating activity of the combinations and Pickering emulsions were also determined using 24-h-old S. aureus P8-AE1 biofilms. All of these treatments exhibited excellent biofilm eradication activity in SCFM2 medium (Fig. 3B and Fig. S2B). Borneol alone at a concentration of 0.5 mg/mL significantly reduced the log CFU/mL from 8.67 to 6.46 (*P *< 0.0001) (see Fig. S5A in the supplemental material). However, not all bacteria within preformed biofilms (24 h) were killed by borneol (0.5 to 4.0 mg/mL). Citral alone at a concentration of 0.5 mg/mL significantly (*P *< 0.0001) reduced the log CFU/mL from 8.84 to 6.55 (Fig. S2B), while citral at 4 mg/mL totally eradicated the preformed biofilms. BC (1:9) at 0.5 mg/mL reduced the number of cells recovered from the biofilm by 3.3 log units, and BC (2:8) at 0.5 mg/mL 100% eradicated the 24-h-old preformed biofilm.

Moreover, Pickering emulsions (0.125 mg/mL) reduced the number of bacteria recovered from the biofilm by at least 1.3 log units. MBEC_90_ values decreased at least 8-fold (0.5 mg/mL to 0.125 mg/mL) compared to those of the the unencapsulated phytochemicals or combinations. The values of MBEC for C-Cap, BC-Cap (1:9), and BC-Cap (2:8) were 1, 1, and 0.5 mg/mL, respectively.

We observed that both planktonic and biofilm S. aureus P8-AE1 are susceptible to low concentrations of antibacterial agents tested in our study, especially Pickering emulsions. Compared to that against P. aeruginosa PAO1, borneol, citral, BC, and Pickering emulsions of these compounds have better antibiofilm efficacy against S. aureus P8-AE1, which is in line with data from a previous study showing that citral is more active against S. aureus (minimum bactericidal concentration [MBC] of 3 mg/mL) than against P. aeruginosa (MBC of >12 mg/mL) (36). Borneol and citral mainly affect the integrity, permeability, and the function of the cytoplasmic membrane of S. aureus and Escherichia coli (37). Membrane-targeting molecules usually do not require an active metabolism of bacteria to exert their bactericidal action, thus resulting in a rapid and effective killing of planktonic bacteria and established biofilms (38). Therefore, borneol and citral may represent attractive templates for persistent biofilm-associated infections.

### Biofilm inhibition in a wound model.

**FIG 4:**
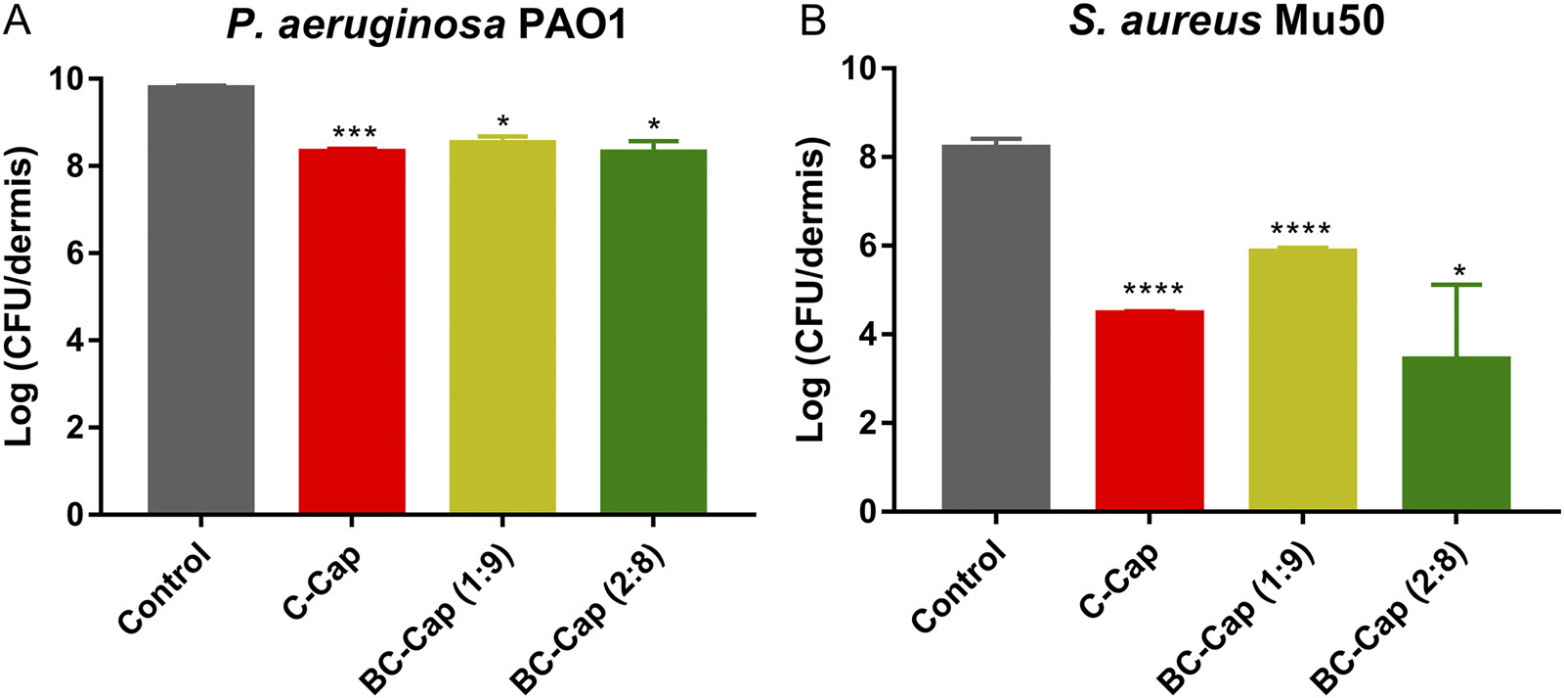
Biofilm inhibitory activity against P. aeruginosa PAO1 and S. aureus Mu50 in the *in vitro* artificial wound model after 24 h of incubation. (A) Control represents untreated bacteria, and Pickering emulsions were tested in a concentration of 10 mg/mL. (B) Control represents untreated bacteria, and Pickering emulsions were tested in a concentration of 1 mg/mL. Error bars represent the standard deviation. *, *P *< 0.05; **, *P *< 0.01; ***, *P* < 0.001; ****, *P *< 0.0001.

The antibiofilm activity toward P. aeruginosa PAO1 and S. aureus Mu50 was also evaluated in a chronic wound model (Fig. 4). For P. aeruginosa, 10 mg/mL borneol, citral, and BC were all dissolved in 6% ethanol, and experiments with 6% ethanol alone were used as control. At a concentration of 10 mg/mL (see Fig. S4A in the supplemental material), borneol and citral alone demonstrated substantial inhibition activity against P. aeruginosa PAO1 biofilm formation, i.e., 5.5- and 5.8-log reduction compared to that of the ethanol control, respectively. When borneol and citral were combined, BC (1:9) at 10 mg/mL displayed the strongest biofilm inhibitory effect, reducing the number of viable bacteria up to 6.59 log units after 24 h of incubation (Fig. 4A), while BC (2:8) at same concentration reduced the number of viable bacteria to 2.6 log units. At 10 mg/mL, Pickering emulsions displayed a significant biofilm inhibitory activity against P. aeruginosa PAO1, reducing the number of viable bacteria up to 1.3 to 1.5 log units after 24 h of incubation (Fig. 4A). Although Pickering emulsions resulted in at least 90% reduction in the CFU count, the biofilm inhibitory activity is far lower than that of the unencapsulated phytochemicals or the combinations (*P *< 0.05, independent samples *t* test).

We also tested borneol, citral, combinations 1:9 and 2:8, and their Pickering emulsions at 1 mg/mL against S. aureus Mu50 (Fig. 4B; see also Fig. S4B). All of these tested treatments except borneol at 1 mg/mL displayed a substantial antibiofilm activity against S. aureus Mu50 in the chronic wound model, reducing the number of the CFU range from a 2.6- to 5.0-log reduction (*P *< 0.05). Importantly, C-Cap, BC-Cap (1:9), and BC-Cap (2:8) at 1 mg/mL resulted in a 3.7-log reduction, 2.4-log reduction, and 4.8-log reduction, respectively, thereby exceeding the activity of citral alone (*P *< 0.05) and BC (2:8) (*P *< 0.001).

Biofilms cause infection, inflammation, and delayed wound healing in 60 to 100% of chronic wounds (39). P. aeruginosa and S. aureus with multidrug resistance are two of the most prevalent organisms isolated from wound sites and are of particular concern due to their elevated levels of antibiotic resistance, rapid growth, and exotoxin production (40–44). Antibiofilm activity of citral, borneol, and relevant Pickering emulsions against clinical isolates P. aeruginosa and S. aureus in different models has been verified in our study. Previous work also showed that citral had the ability to reduce bacterial adherence, and sequential exposure of these pathogens to increasing concentrations of citral did not pose a risk of resistance development in the adapted cells of wound or clinical isolates, including S. aureus and methicillin-resistant S. aureus (MRSA) (45). Except for their antibacterial activity, anti-inflammatory and proliferative qualities of citral and borneol are also useful in the treatment of infected wounds (46). For example, the incorporation of borneol and chitosan film accelerated the wound-healing process in an excision wound model by improving inflammatory profile (47). Citral loaded in ZnO/chitosan nanoparticles indicated better antibacterial activities against P. aeruginosa and S. aureus *in vitro* and *in vivo* than citral alone and sped up wound healing in mice by altering the expression of inflammatory and proliferative genes (48, 49). Therefore, the incorporation of borneol and citral as well as relevant Pickering emulsions have the potential to heal wound infections.

### Effect of citral and Pickering emulsions on the survival of G. mellonella larvae infected with S. aureus P8-AE1.

**FIG 5:**
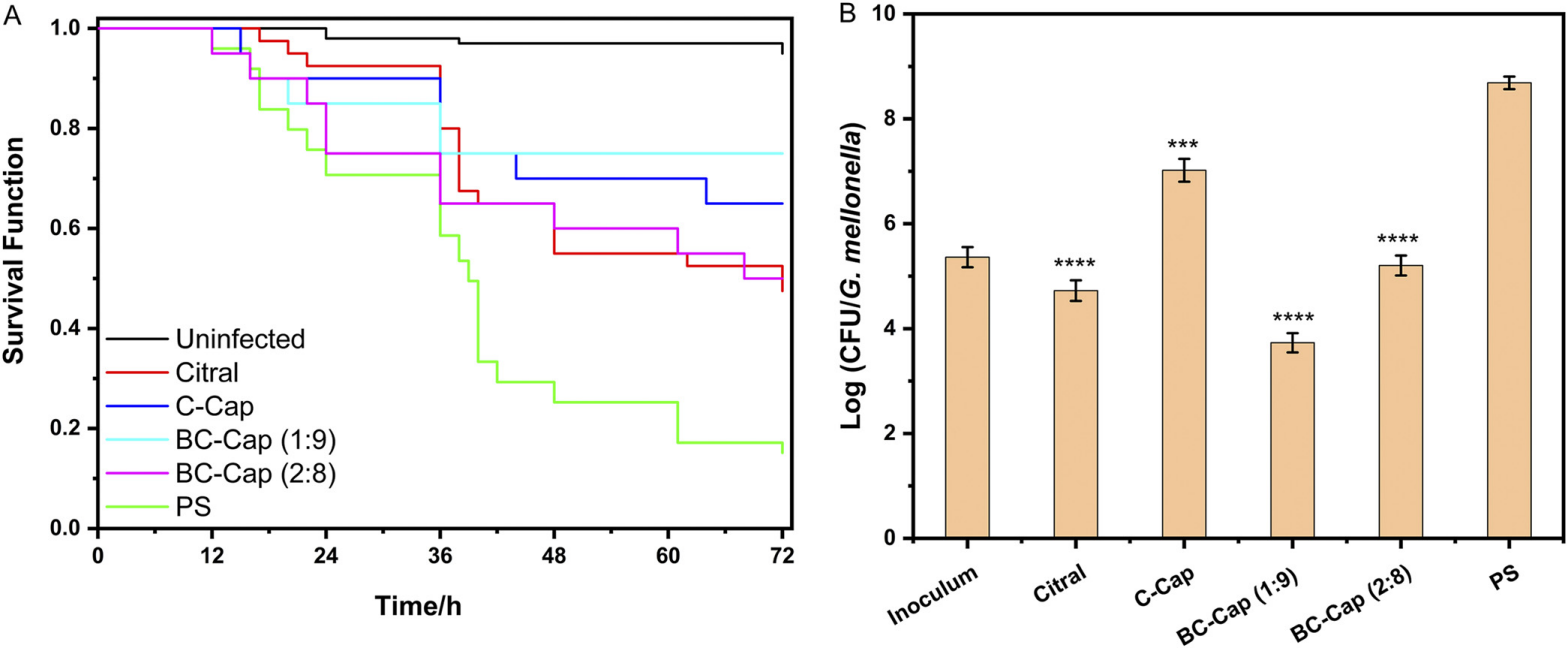
Kaplan-Meier survival curve of infected G. mellonella larvae and bacterial burden during infection. (A) Survival of G. mellonella larvae infected with S. aureus P8-AE1 after various treatments (1 mg/mL citral, 1 mg/mL C-Cap, 2 mg/mL BC [1:9] and BC [2:8], 0.5 mg/mL BC-Cap [1:9], and 1 mg/mL BC-Cap [2:8]). (B) S. aureus P8-AE1 burden after infection and treatment followed by 72 h of incubation. ***, *P *< 0.001; ****, *P* < 0.0001.

To further explore their *in vivo* toxicity and antibacterial activity of these phytochemicals and relevant Pickering emulsions, a G. mellonella infection model was then exploited in our study. The tested concentrations for citral, BC (1:9), BC (2:8), C-Cap, BC-Cap (1:9), and BC-Cap (2:8) were 1, 2, 2, 1, 0.5, and 1 mg/mL, respectively, and at these concentrations, citral, BC, C-Cap, and BC-Cap did not result in significant death, indicating that the treatments were not toxic toward the larvae (Fig. S5). Doses of about 2 × 10^5^ CFU/larvae were selected for further experiments with S. aureus P8-AE1 since they showed significant differences (*P *< 0.001) in larval survival compared to that of the uninfected control group and a gradual reduction in survival rate covering the whole experimental period (Fig. 5A). We first excluded the effect of ethanol on the survival of infected bacteria (Fig. S5A to C). After infection with S. aureus P8-AE1, survival curves showed that the groups of larvae treated with citral (*P = *0.01), C-Cap (*P = *0.0004), BC-Cap (1:9) (*P = *0.0002), and BC-Cap (2:8) (*P = *0.008) demonstrated an increase in survival compared to that of the negative control group (Fig. S5A and D to F). In parallel, bacterial counts in all of the treated group were significantly lower (*P *< 0.001) than those in the control group (physiological saline [PS] treated) (Fig. 5B). The 0.5 mg/mL BC-Cap (1:9) had a better effect than citral (*P *< 0.0001), C-Cap (*P *< 0.0001), and BC-Cap (2:8) (*P* < 0.0001) at the concentration of 1 mg/mL.

The advantages of using the G. mellonella model are numerous, including low cost and easy maintaining and handling, allowing precise quantification of the inoculum etc., and the most important point is that G. mellonella are not affected by the legal or ethical constraints that restrict the use of mammals (50, 51). Therefore, G. mellonella is well accepted by the scientific community worldwide as an *in vivo* and preclinical model to screen novel antibacterial agents (52, 53). A similar experiment was also carried out by other researchers to test the effect of cinnamaldehyde on bacterial virulence. Both cinnamaldehyde and citral belong to plant-derived aldehydes. *In vivo* testing of cinnamaldehyde in G. mellonella larvae infected with S. aureus showed that cinnamaldehyde improves larvae survival while diminishing bacterial load in their hemolymph (54), which is consistent with our findings about citral. As a consequence, G. mellonella can be exploited to assess the virulence of microbial pathogens and produce results about novel antibacterial agents.

### Conclusions.

Combination of phytochemicals and encapsulation in Pickering emulsion are promising strategies to improve the efficacy of citral, especially against biofilms under challenging host-mimicking conditions. The results of the present study underlined the importance of evaluating the biological properties of phytochemicals in *in vivo*-like model systems representative of specific infectious sites in order to make a more realistic prediction of their therapeutic success and avoid the inclusion of unpromising antibacterial agents in animal studies and clinical trials.

## MATERIALS AND METHODS

### Bacterial strains, growth conditions, and chemicals.

Overnight cultures of P. aeruginosa PAO1 (wound isolate), S. aureus P8-AE1 (CF sputum), and S. aureus Mu50 (wound isolate) were routinely prepared by inoculating from −80°C frozen stock into tryptic soy broth (TSB) (Lab M, Moss Hall, UK). Pure cultures and serially 10-fold diluted bacterial solutions were cultured on tryptic soy agar (TSA) (Lab M, Moss Hall, UK). Mannitol salt agar (MSA) (selective medium for S. aureus) was purchased from Sigma-Aldrich (Bornem, Belgium). Milli-Q water was used for all experiments.

Borneol (≥98% purity) was obtained from Guangdong Huaqingyuan Biological Technology Co., Ltd (Meizhou, China), and citral (98% purity) was purchased from Aladdin Biochemical Technology Co., Ltd (Shanghai, China). Pickering emulsions (C-Cap and BC-Cap) were prepared as described previously (4) to improve their dispersion of citral and the combination of borneol and citral (BC) in different physiologically relevant fluid environments and thereby further improve their antibiofilm efficacy. They were stored at 4°C before use. Since the maximum solubility of borneol in citral was ca. 28.35 g/100 g (4), we first dissolved the highly hydrophobic solid borneol into citral at a mass ratio of 1:9 or 2:8 prior to emulsification. SiO_2_-NH_2_ NPs were then suspended in pure Milli-Q water (pH adjusted to 10 with 1 M NaOH solution) to deprotonate the surface amines and promote covalent attachment to citral. The oil phase (citral or BC, 10% vol/vol) was subsequently added to the above aqueous phase and homogenized at 24,000 rpm for 2.5 min by Miccra D-1 high-shear rotor homogenizer (Miccra GmbH, Heitersheim, Germany). The emulsion was further homogenized by ultrasonication (Kunshan Ultrasonic Instruments, Kunshan, China) for 100 s at 40% power. The resulting oil/water emulsions were allowed to react for 1 day before use.

### Biofilm inhibition in SCFM2.

Stock solutions (100 mg/mL in 60% sterile ethanol) of borneol, citral, and borneol-citral combinations (BC) in ratios 1:9 and 2:8 were prepared and diluted to the required concentrations with sterile SCFM2. C-Cap/BC-Cap were prepared at 80 mg/mL and diluted to the required concentrations with sterile SCFM2. SCFM2 was prepared as described previously with minor modifications (55). Mucin was autoclaved at 121°C for 20 min prior to adding buffered base. The final pH value of SCFM2 was adjusted to 6.8 using NaOH (1 M solution) before use. The minimum biofilm inhibitory concentrations (MBICs) of phytochemicals and Pickering emulsions were determined by plate counts as described previously (55). Briefly, a 2-fold dilution series of phytochemicals or Pickering emulsions were prepared in SCFM2; concentrations tested ranged from 0.1563 to 20 mg/mL (for P. aeruginosa PAO1) and 0.125 to 4 mg/mL (for S. aureus P8-AE1). The optical density at 590 nm (OD_590_) of the overnight culture was measured in TSB and diluted in SCFM2 to a density of approximately 5 × 10^5^ CFU/mL. This suspension was added to flat bottom 96-well plates (100 μL/well) together with 100 μL of the diluted antimicrobial agent. After 24 h of incubation at 37°C, cells were harvested after vortexing and sonicating (2 times, 5 min for each), and the number of CFU in the biofilms was determined by making a 10-fold serial dilution series and plating. Suitable negative controls containing no antimicrobial and/or no bacteria were included on every plate. The MBIC was defined as the lowest concentration of antibacterial agent that inhibited biofilm growth by at least 90% compared to that of the untreated control (55, 56). All experiments were performed in biological triplicates (with at least three technical replicates in each biological replicate).

### Biofilm eradication in SCFM2.

An overnight culture was diluted and resuspended in SCFM2 to a density of approximately 5 × 10^5^ CFU/mL. This bacterial suspension (100 μL) was added to a flat-bottom 96-well plate and incubated at 37°C for 24 h. After 24 h, these biofilm aggregates were exposed to 100 μL phytochemicals or Pickering emulsions for an additional 24 h as described previously (57). After an additional 24 h of incubation at 37°C, cells were harvested after vortexing and sonicating (2 times, 5 min each), and the number of CFU was determined by plating on the TSA plate (58). The minimal biofilm eradication concentration (MBEC) was defined as the lowest concentration that prevents visible growth on the TSA plate after 24 h of incubation recovered from preformed biofilm (56, 59). The MBEC_90_ was defined as the lowest concentration of antibacterial agent that killed at least 90% bacteria in preformed biofilm (1-log-unit difference) compared to that in the control. All experiments were performed in biological triplicates (with at least three technical replicates in each biological replicate).

### Biofilm inhibition in the artificial wound model.

The biofilm wound model has been described before (60, 61). A spongy artificial dermis (upper layer, chemically cross-linked hyaluronic acid; lower layer, hyaluronic acid and collagen) was used as a substrate for biofilm formation to mimic biofilm formation at the air-liquid interface in real wounds. Each sheet of artificial dermis was placed in the well of a 24-well microtiter plate. A mixed medium of lyophilized bovine plasma (Sigma), 19 mL of sterile Bolton broth, 1 mL of horse blood, and 10 U of heparin (20 μL of a 100 kU solution) was prepared. An appropriate volume of the above medium was added on and around the dermis to keep the whole dermis moist. The dermis was then inoculated with 10 μL of an overnight culture adjusted to 1 × 10^6^ CFU/mL (P. aeruginosa PAO1 and S. aureus Mu50). Pickering emulsions were added to the top of the infected dermis. After 24 h of incubation at 37°C, the dermis was washed and transferred into 9 mL of 0.9% physiological saline (PS). Biofilm cells on the dermis were collected by two alternating cycles of sonication (5 min) and vortexing (5 min). The number of CFU/dermis was quantified by plating. Three independent experiments were performed with three replicates each.

### Evaluation of toxicity and antimicrobial activity in the G. mellonella infection model.

G. mellonella larvae were purchased from local fishing shops (Gent, Belgium). Larvae were stored in wood shavings in the dark at 13°C before use. Healthy larvae were identified by a uniform creamy color, with no indications of melanization such as black spots or markings. At least 10 randomly selected G. mellonella larvae (weight range, 200 to 300 mg) were used per experimental group in all assays. Experiments were performed with strain S. aureus P8-AE1. For survival analyses, overnight culture of S. aureus P8-AE1 strain was centrifuged (5,000 rpm, 5 min) and resuspended twice in sterile PS. Phytochemicals or Pickering emulsions were diluted with the same volume of the above bacterial suspension to give the desired concentrations. Then 10-μL doses of diluted bacterial suspensions (2 × 10^5^ CFU/G. mellonella) with treatments together were quickly (less than 2 min per experimental group to avoid bacterial death) injected into the right hind proleg of the larvae using a Hamilton syringe (Hamilton Company, Reno, NV, USA), and the infected larvae were incubated in the dark without food or water at 37°C for up to 72 h. The following control groups were included: larvae inoculated with PS, larvae inoculated with ethanol dissolved in PS, and larvae inoculated with S. aureus P8-AE1 only, respectively. Larvae were recorded as dead when they met the following criteria (62): (i) complete melanization, (ii) did not respond to touch, and (iii) could not correct itself when rolled onto its back. Experiments were repeated at least 3 times (at least 10 larvae per group). The dead larvae were immediately transferred to a 50-mL sterile centrifuge tube, homogenized, and vortexed, and the bacterial number of CFU in the larvae were quantified by making a 10-fold serial dilution series and plating on MSA.

### Statistical analysis.

All data are presented as mean ± standard deviation (SD). Comparisons between multiple groups were performed using two-way analysis of variance (ANOVA) followed by a Bonferroni test (GraphPad Prism version 7.00; GraphPad Software, La Jolla, CA, USA); comparisons between different concentrations were performed using one-way ANOVA followed by a Tukey test. Independent sample *t* tests were used to evaluate differences between the two treatment groups. Statistical significance was assessed at the 95% confidence level. Survival analysis and statistical significance were determined using the log rank test and the Kaplan-Meier survival curves (OriginPro 2021; OriginLab Corp., Northampton, MA). Significance level in G. mellonella bacterial burden assay was analyzed by independent sample *t* tests.
