# Supplementary material for: Antibiofilm Activities of Borneol-Citral-Loaded Pickering Emulsions against Pseudomonas aeruginosa and Staphylococcus aureus in Physiologically Relevant Chronic Infection Models
Source: Microbiol Spectr. 2022 Oct 4;10(5):e01696-22. doi: 10.1128/spectrum.01696-22 (PMC9602683; doi:10.1128/spectrum.01696-22)
Supplement: Supplemental file 1 — Fig. S1 to S5. Download spectrum.01696-22-s0001.pdf, PDF file, 0.3 MB [file spectrum.01696-22-s0001.pdf]

Supplementary Figures

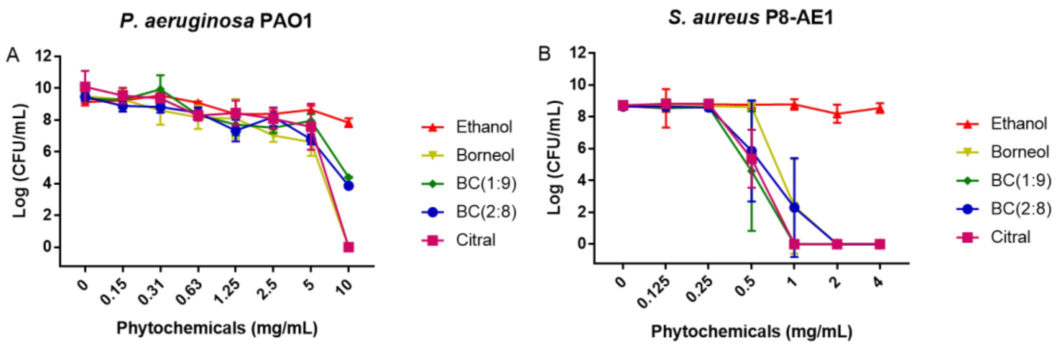

Figure S1 Effect of phytochemicals on biofilm inhibition of *P. aeruginosa* PAO1 and *S. aureus* P8-AE1 in SCFM2. Error bars represent the standard deviation.

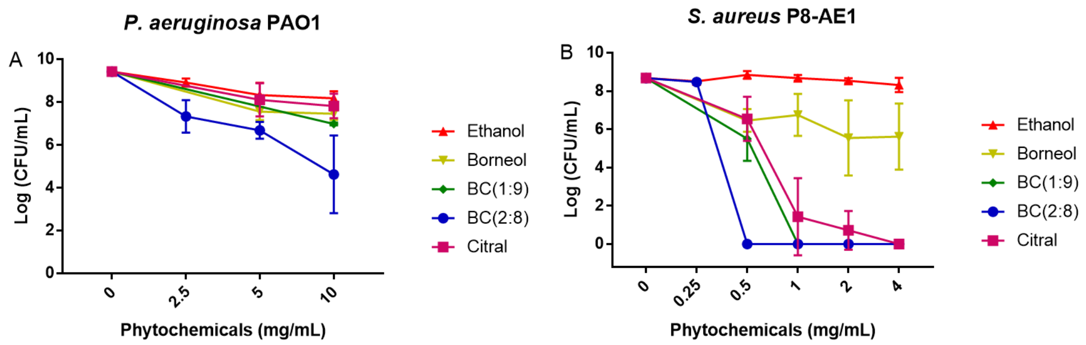

Figure S2 Number of culturable cells (CFU/mL) recovered from a 24-h biofilm grown in SCFM2 after exposure to different concentrations of borneol, citral, or a combination of both. Error bars represent the standard deviation.

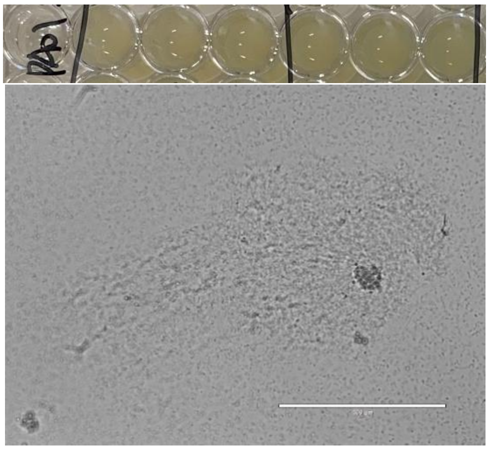

Figure S3 Microscopic observation of *P. aeruginosa* PAO1 biofilms after 24-h of incubation

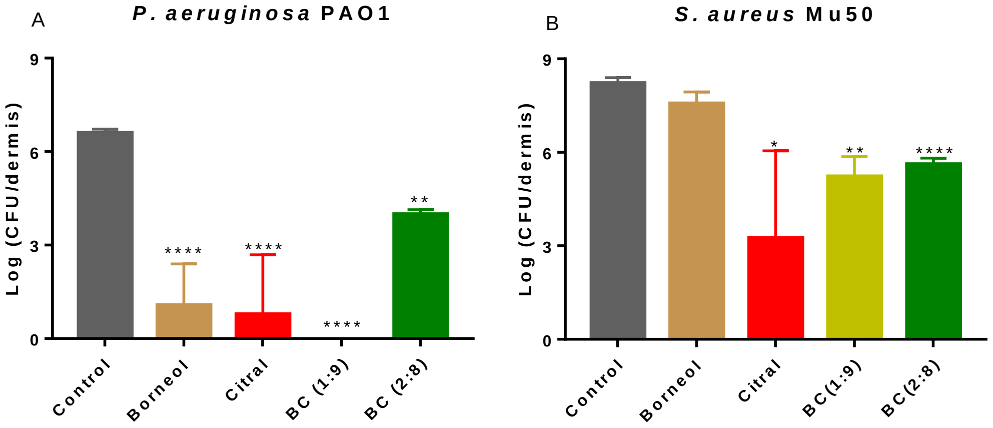

Figure S4 Biofilm-inhibitory activity against *P. aeruginosa* PAO1 and *S. aureus* Mu50 in the *in vitro* artificial wound model after 24 h of incubation. (A) Control represents 6% ethanol and phytochemicals were tested in a concentration of 10 mg/mL； (B) Control represents 0.6% ethanol and phytochemicals were tested in a concentration of 1 mg/mL. Error bars represent the standard deviation. *, $P < 0.05$; **, $P < 0.01$; ***, $P < 0.001$ and ****, $P < 0.0001$.

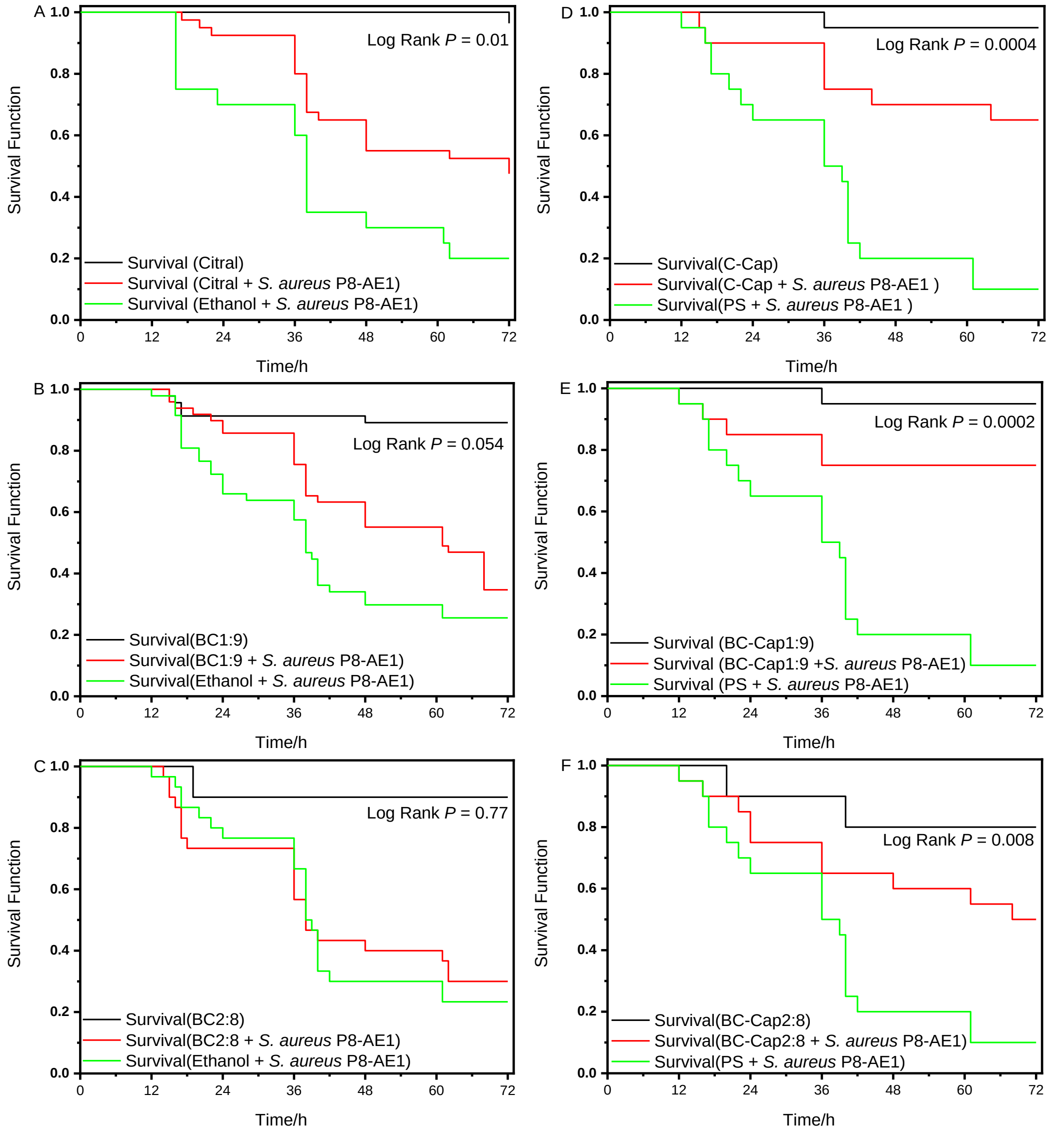

Figure S5 Kaplan-Meier survival-curve of infected *G. mellonella* larva treated with different phytochemicals and Pickering emulsions. Survival of *G. mellonella* larvae infected with *S. aureus* P8-AE1 after treated with 1 mg/mL citral (A), 2 mg/mL BC (1:9) (B) and BC (2:8) (C), 1 mg/mL C-Cap (D),0.5 mg/mL BC-Cap (1:9) (E) and 1 mg/mL BC-Cap (2:8) (F), respectively.
